# Association between enuresis and obesity in children with primary monosymptomatic nocturnal enuresis

**DOI:** 10.1590/S1677-5538.IBJU.2018.0603

**Published:** 2019-09-02

**Authors:** Yanli Ma, Ying Shen, Xiaomei Liu

**Affiliations:** 1 Department of Nephrology, Beijing Children’s Hospital, Capital Medical University, National Center for Children’s Health, China;; 2 Beijing Key Laboratory of chronic kidney disease and blood purification of children, South Lishi Road, Xicheng District, Beijing, China

**Keywords:** Nocturnal Enuresis, Behavior Therapy, Treatment Failure

## Abstract

**Objective:**

The purpose of this study was to determine whether the presence of obesity was related with symptoms of nocturnal enuresis (NE) and the efficacy of behavioral intervention in the treatment of NE.

**Materials and Method:**

The patients diagnosed with primary monosymptomatic nocturnal enuresis (PMNE) were studied retrospectively. NE severity was classified as mild, moderate, and severe according to the frequency of enuresis. The children were divided into three groups, namely normal weight (5th-84th percentile), overweight (85th-94th percentile), and obesity (≥95th percentile), according to their Body Mass Index (BMI) percentage. The relationship between obesity level and enuresis severity was analyzed. After three months of behavioral therapy, the efficacy of treatment among normal, overweight, and obese groups were evaluated. Moreover, the predictive risk factors for treatment failure were investigated.

**Results:**

The rates of severe enuresis in patients with normal weight, overweight, and obesity were 63.9%, 77.5%, and 78.6%, respectively. Obese children depicted higher odds of having severe enuresis compared with normal-weight children (OR: 1.571; 95% confidence interval [CI]: 1.196-2.065; P=0.001). The odds of presenting with severe enuresis were 1.99 times higher in children who are obese or overweight compared to children with normal weight (OR: 1.994; 95% CI: 1.349-2.946; P=0.001). The complete response of the normal group was higher than those of the overweight and obese groups (26.8% vs. 14.0%, P=0.010; 26.8% vs. 0.0%, P=0.000). Overweight children showed higher complete response than obese ones (14.0% vs. 0.0%, P=0.009). Logistic regression analysis revealed that obesity level and enuresis frequency were significantly related to the treatment failure of behavioral intervention.

**Conclusions:**

Obesity is associated with severe enuresis and low efficacy of behavioral therapy in children with nocturnal enuresis.

## INTRODUCTION

Nocturnal enuresis (NE) is the most common pediatric urological developmental disorder (1). The prevalence of enuresis is up to 20% in children aged 5 years old, and severe enuresis can persist indefinitely with prevalence rate of 2%-3% in adulthood (2, 3). Primary monosymptomatic nocturnal enuresis (PMNE) occurs in children who have previously been dry for <6 months without any (other) lower urinary tract symptoms or a history of bladder dysfunction (4). Although many different underlying pathophysiological mechanisms have been proposed to explain NE, its etiology remains unclear (5-8).

Obesity is a common and growing problem worldwide. According to Freedman et al., childhood obesity negatively affects blood pressure, lipid profiles, glucose metabolism, and cardiovascular diseases (9). Obese children are more likely affected by some other health problems, such as respiratory diseases, infertility, degenerative joint diseases, proteinuria, depression, anxiety, and discrimination in social life and the workplace, as compared with non-obese ones (10).

Obesity is related to the prevalence of enuresis and the treatment response of enuresis (11-13). However, analysis on the relationship between the symptoms of enuresis and obesity is lacking. In addition, the relations between enuresis and obesity have been controversial, including the association between body mass index (BMI) and therapeutic efficacy in the treatment of enuresis. According to several scholars, obesity and NE are not associated (14-16). Hence, further research evaluating the association between obesity and enuresis is needed to understand the possible role of obesity in the pathogenesis and treatment responses of enuresis.

This study aims to assess the association between obesity and enuresis, including the relationship of symptom classification of enuresis and the treatment responses of behavioral therapy for obese enuretic patients. The relationship between treatment failure and possible risk predictive variables is also analyzed.

## MATERIALS AND METHODS

### Participants and methods

This study was approved by the ethics committee of Beijing Children’s Hospital and was designed in accordance with the Declaration of Helsinki. The participants were children and adolescents who were referred from the enuresis outpatient clinic in Beijing Children’s Hospital between May 2016 and December 2017 and who had been diagnosed with PMNE. Consent forms were signed by their parents or caregivers. The inclusion criteria were as follows: PMNE without previous treatment of any kind during the past 6 months, aged between 5 and 15 years old, and signed informed consent. Exclusion criteria were defined as known anatomical, urological, gastrointestinal, cardiovascular, endocrinological, and/or neurological pathologies, and abnormalities in abdominal ultrasound or urinalysis that would interfere with evaluation. Demographic and disease characteristics, including age, gender, weight, height, family history of enuresis, frequency of enuresis, daytime lower urinary tract symptoms (such as urinary urgency, urinary frequency, and daytime urinary incontinence), and any treatment for NE were assessed by a questionnaire administered to the parents. After history taking and physical examination, all participants underwent urine analysis, urine culture, and urinary tract ultrasound. Patients with abnormal results were excluded. NE severity was categorized as mild, moderate, and severe according to the frequency of enuresis. NE every night or 5-6 wet nights a week was defined as severe, 1 or 2 days a week was considered mild, and somewhat in between was regarded as moderate.

BMI was calculated using the formula weight/height^2^ (kg/m^2^). In this study, the standardized growth curve of Chinese children and adolescents aged 0-18 years established by Li et al. was used (17). The children were divided into three groups, namely normal weight (5th-84th percentile), overweight (85th-94th percentile), and obesity (≥95th BMI percentile), according to their BMI percentage (18).

Behavioral intervention is the first line of treatment and pharmacotherapy should not be initiated in children unless nonpharmacological interventions have failed. Behavioral intervention strategies involve educating families regarding enuresis and its treatment, offering suggestions for voiding patterns and frequency, limiting fluid intake, carrying the child to the toilet at night or waking the child up for urination, providing daily motivation and exercises aimed at increasing bladder capacity, and treating constipation when present. The child must have an active role in bed cleaning after bed-wetting, should never be punished for wetting his/her bed, and must be rewarded if he/she reached the goal, as monitored by the signed wet and dry nights in a calendar. All patients were required to record daytime and overnight bladder diaries. If the patients experienced any difficulty, they sought help from their parents. We evaluated the efficacy of treatment in 3 months among the three groups after the start of behavioral therapy on the basis of the voiding diary kept by patients or their parents. Night-time urine volume was calculated as the weight of diapers plus the first voided volume in the morning. The presence of nocturnal polyuria was defined as an average night-time urine volume exceeding 130% of expected bladder capacity (30 + [age in years × 30] mL) (4, 19). The treatment response was defined by the 2014 ICCS criteria as follows: no response, partial response (<50% reduction of wet nights, 50%-99% reduction in the frequency of NE), and complete response (100% reduction of enuresis episodes) (20). Moreover, the predictive risk factors for treatment failure (partial response and no-response) were investigated.

### Statistical analysis

Statistical analyses were performed using the SPSS 19.0 software (SPSS Inc., Chicago, IL, USA). Univariate logistic regression and Pearson chi-square test were conducted to investigate the possible relationship between enuresis severity and obesity level. Enuretic patients were stratified by age, and chi-square test was then used to compare the differences in the treatment responses among patients aged 11-15 years and 5-10 years. Multivariable logistic regression model was performed to identify risk factors among age, gender, frequency of enuresis, degree of obesity, family history of enuresis, presence of nocturnal polyuria, and treatment failure. A P value less than 0.05 was considered statistically significant.

## RESULTS

A total of 666 patients with PMNE were included in this study. Demographic information of these participants is shown in Table-1. The children were aged 5-14.5 years with a median age (interquartile range) of 6.5 (5.1-9.1) years. No significant differences on age, gender, family history of enuresis, and nocturnal polyuria were found among normal, overweight, and obese groups (Table-1).

**Table 1:** Baseline clinical characteristics and demographic features.

Variables	Normal (n, %)	Overweight (n, %)	Obese (n,%)	Total (n)	P value
**Gender**					
Male	248 (52.1%)	67 (55.8%)	34 (48.6%)	349	0.608
Female	228 (47.9%)	53 (44.2%)	36 (51.4%)	317	
**Age (years)**					
5-9	356 (74.8%)	78 (65.0%)	54 (77.1%)	488	0.071
10-15	120 (25.2%)	42 (35.0%)	16 (22.9%)	178	
**Family history of NE**					
No	337 (70.8%)	83 (69.2%)	41 (58.6%)	461	0.118
Yes	139 (29.2%)	37 (30.8%)	29 (41.4%)	205	
**Nocturnal polyuria**					
Present	265 (55.7%)	56 (46.7%)	41 (58.6%)	362	0.158
Absent	211 (44.3%)	64 (53.3%)	29 (41.4%)	304	

A total of 214 patients (32.2%) had mild-moderate NE, and 452 (67.8%) had severe NE. The rates of severe enuresis in patients with normal weight, overweight, and obesity were 63.9%, 77.5%, and 78.6%, respectively. The rate of severe enuresis in patients with normal weight was lower than that in overweight children, and the difference was statistically significant (P=0.005, adjusted P-value=0.0125). No statistically significant differences were found in the rate of severe enuresis between obese and overweight groups and between obese and normal groups (P=0.864; P=0.015). The relationship between NE severity and the presence of obesity is shown in Table-2. Obese children depicted higher odds of having severe enuresis compared with normal-weight children (OR: 1.571; 95% confidence interval [CI]: 1.196-2.065; P=0.001). The odds of presenting with severe enuresis were 1.99 times higher in children who were obese or overweight compared to children with normal weight (OR: 1.994; 95% CI: 1.349-2.946; P=0.001).

**Table 2:** The relationship between enuresis severity and the presence of obesity.

Groups	Mild-moderate n (%)	Severe n (%)	Total n (%)	X^2^	P Value
Normal n (%)	172 (36.1%)	304 (63.9%)	476 (100.0%)	12.279	0.002
Overweight n (%)	27 (22.5%)	93 (77.5%)	120 (100.0%)		
Obese n (%)	15 (21.4%)	55 (78.6%)	70 (100.0%)		

Among the 666 patients, 48 did not follow the doctor’s advice, 32 did not regularly consult a doctor, and 28 were lost to follow-up. Thus, a total of 558 participants completed this treatment phase. The complete response of the normal group was higher than those of the overweight and obese groups, and the differences were statistically significant (26.8% vs. 14.0%, P=0.010; 26.8% vs. 0.0%, P=0.000). Overweight children showed higher complete response than obese ones, and the difference was statistically significant (14.0% vs. 0.0%, P=0.009). Among the 5-to 10-year-old patients, the complete response of the obese group was lower than that of the normal group (P=0.000); however, no significant differences were found regarding complete response between the normal and overweight groups and between the overweight and obese groups (P=0.045, P=0.019, adjusted P-value=0.0125). Among the 11- to 15-year-old patients, the complete response of the normal group was higher than that of the overweight group (P=0.007). However, no statistically significant differences were observed in the complete response between the normal and obese groups and between the overweight and obese groups (P=0.026, P=0.016, adjusted P-value=0.0125, Table-3).

**Table 3:** The treatment responses of behavioral therapy.

Groups		Nonresponse n (%)	Partial Response n (%)	Complete Response n (%)	X^2^	P Value
5-15 years	Normal n (%)	67 (17.4%)	214 (55.7%)	103 (26.8%)	28.314	0.000
	Overweight n (%)	18 (15.8%)	80 (70.2%)	16 (14.0%)		
	Obese n (%)	12 (20.0%)	48 (80.0%)	0 (0.0%)		
5-10 years	Normal n (%)	46 (16.0%)	164 (56.9%)	78 (27.1%)	22.205	0.000
	Overweight n (%)	16 (21.6%)	48 (64.9%)	10 (13.5%)		
	Obese n (%)	8 (17.0%)	39 (83.0%)	0 (0.0%)		
11-15 years	Normal n (%)	21 (21.9%)	50 (52.1%)	25 (26.0%)	14.071	0.001
	Overweight n (%)	2 (5.0%)	32 (80.0%)	6 (15.0%)		
	Obese n (%)	4 (30.8%)	9 (69.2%)	0 (0.0%)		

Multivariable logistic regression analysis of predictive risk factors for treatment failure.

A logistic regression model was used to investigate the relationship between treatment failure and possible risk predictive variables by using six factors (age, gender, frequency of NE, family history of NE, presence of nocturnal polyuria, and obesity) as independent variables and treatment failure as a dependent variable (Table-4). Logistic regression analysis revealed that obesity level (OR: 2.633, 95% CI: 1.615-4.291) and enuresis frequency (OR: 3.350, 95% CI: 2.082-5.390) were significantly related to the treatment failure of behavioral therapy.

**Table 4:** Logistic regression analysis on therapeutic effect of behavioral therapy.

Factors	B	Standard error	Wald value	P value	OR	95% confidence interval	

						Lower limit	Upper limit
Gender	0.093	0.233	0.159	0.690	1.097	0.694	1.734
Age	0.032	0.276	0.013	0.909	1.032	0.600	1.774
Enuresis frequency	1.209	0.243	24.816	0.000	3.350	2.082	5.390
Family history of NE	-0.428	0.237	3.263	0.071	0.652	0.410	1.037
Nocturnalpolyuria	-0.001	0.218	0.000	0.995	0.999	0.651	1.531
Obesity level	0.968	0.249	15.075	0.000	2.633	1.615	4.291

## DISCUSSION

NE has been found to be related to obesity. Overweight and obese children tend to consume an unhealthy diet that may overwhelm their functional bladder capacity and result in NE (21, 22). Weintraub et al. reported that NE affects 9% of children with normal weight, 16% of overweight children, and 30% of obese children and adolescents (7-18 years old) (12). Their results also showed that the odds ratio of enuresis in obese children was 6.5 folds more than that in normal weight children. Guven et al. and Sally et al. analyzed the effect of obesity on the treatment responses of enuresis, but their conclusions were contradictory (13, 16). This finding led us to investigate a possible association between obesity and enuresis in children and adolescents. In the present study, we examined the relationship between enuresis severity and obesity degree, which has not been explored in previous studies.

In our study, children who are obese and overweight were more likely to develop severe enuresis compared with those with normal weight. Some common pathogenesis for obesity and enuresis may explain this result. First, obesity exposes the pelvic floor to elevated intra-abdominal and intra-vesical pressure, thereby compromising the functional bladder capacity (23). This reduced functional bladder capacity plays an important role in the pathogenesis of enuresis. Second, monosymptomatic NE occurs after psychological stress or trauma and results in increased psychological distress for the child (24). Obese children are also under higher psychological distress than their non-obese peers (25). Obesity and enuresis are closely related to psychological factors, indicating that several shared mechanisms may be involved in their pathogenesis. Third, obesity is associated with hyperglycemia, which can cause diuresis and lower urinary tract symptoms. Fourth, adolescent obesity is related with sleep-disordered breathing (SDB) conditions, such as habitual snoring, obstructive sleep apnea (OSA), upper airway resistance syndrome, and hypoventilation (26-28). SDB is directly related to NE. Habitual snorers are at a greater risk of having NE than non-snorers (29). Moreover, bedwetting is predictive of OSA in children (30). The association between SDB and enuresis may be explained by large amounts of overnight sodium and urine excretion, which are probably caused by the increased secretion of atrial natriuretic peptide in patients with SDB (31). Thus, explaining the association between obesity and severe enuresis is not difficult.

In this study, the incidence of complete response was lower in overweight and obese patients than in normal-weight enuretic patients. Moreover, the level of obesity (OR: 2.633, 95% CI: 1.615-4.291) was an independent risk factor for the treatment failure of behavioral therapy. This result was consistent with previous studies. Guven et al. observed a good response to standard treatment in patients with a BMI below the 85th percentile (13). They speculated that the low treatment success rates in patients with high BMI suggest that obesity and incontinence may share a common etiology. Obesity is related to hormonal abnormalities in some children, and NE may be associated with an abnormality of antidiuretic hormone secretion (10, 32). Hence, pituitary or other central nervous system abnormality could be the cause of both conditions. Kovacevic et al. also reported that the low response rate in patients with NE is associated with obesity (33). Obese children often suffer from SDB, which is closely correlated with NE (34). The association between enuresis and SDB in children is supported by the decrease in enuresis frequency or even by the complete resolution of enuresis after the successful treatment of SDB (35). Although we did not evaluate this condition in our study, obesity may have increased the NE via the mechanism of SDB and negatively affected the treatment outcomes.

In our study, multivariable logistic regression analysis revealed that enuresis frequency (OR: 3.350, 95% CI: 2.082-5.390) was significantly related to the treatment failure of behavioral therapy. This result is in line with previous findings. Kurt et al. reported that NE frequency is a predictive factor in estimating the effectiveness of behavioral treatment (36). In their study, nearly half of the patients who had all days or 5-6 days of enuresis in a week did not show any response to behavioral interventions. Similarly, Önol et al. showed that NE severity is an independent risk predictor of complete response (37).

In view of the above findings of this study, some suggestions may be helpful in guiding clinical practice. First, although losing weight is fraught with difficulties and challenges, weight control should be attempted as the initial step in obese children with NE. Second, obesity is associated with an unhealthy diet. Ferrara et al. reported that specific dietary advices can effectively manage PMNE (38). Thus, dietary recommendations may be necessary for enuretic children with obesity. Third, a previous study suggested that obesity is associated with a low rate of voiding diary completion (13). In this case, behavioral intervention, including alarm therapy, may be prone to failure. Pharmacological intervention might be appropriate. Fourth, several medical conditions, such as SDB, psychopathological disorders, and type 2 diabetes mellitus, co-occur at increased rates among obese and enuretic children. Therefore, the active treatment of comorbidity may be beneficial to the remission of both in some extent.

The limitations of this study are as follows: First, risk factors affecting treatment response, which are indicators for detrusor overactivity (e.g., bladder wall thickness, and bladder volume), have not been analyzed and thus must be investigated in the future. Second, this study reveals an association between enuresis and obesity, but does not prove the cause and effect. Third, the duration of behavioral therapy is only for 3 months. Further study is needed to clarify the role of obesity in the efficacy of long-term behavioral intervention.

In conclusion, obesity is associated with severe enuresis and low efficacy of behavioral therapy in children with NE. Obesity should be considered in enuretic patients, especially if they display severe symptoms of enuresis or they fail to respond to behavioral therapy.

## References

[1] 1. Seibold J, Alloussi S, Todenhöfer T, Stenzl A, Schwentner C. [Primary monosymptomatic enuresis: diagnostics and therapy]. Urologe A. 2013;52:9-10, 12-4. 10.1007/s00120-012-3074-4 23292255

[2] 2. Weaver A, Dobson P. Nocturnal enuresis in children. J Fam Health Care. 2007;17:159-61. 17990655

[3] 3. Yeung CK, Sihoe JD, Sit FK, Bower W, Sreedhar B, Lau J. haracteristics of primary nocturnal enuresis in adults: an epidemiological study. BJU Int. 2004;93:341-5. 10.1111/j.1464-410x.2003.04612.x 14764133

[4] 4. Nevéus T, von Gontard A, Hoebeke P, Hjälmås K, Bauer S, Bower W, et al. The standardization of terminology of lower urinary tract function in children and adolescents: report from the Standardisation Committee of the International Children’s Continence Society. J rol. 2006;176:314-24. 10.1016/S0022-5347(06)00305-3 16753432

[5] 5. Wolfe-Christensen C, Fedele DA, Grant D, Veenstra AL, Kovacevic LG, Elder JS, et al. Factor analysis of the pediatric symptom checklist in a population of children with voiding dysfunction and/or nocturnal enuresis. J Clin Psychol Med Settings. 2014;21:72-80. 10.1007/s10880-013-9375-y 24158241

[6] 6. Abou-Khadra MK, Amin OR, Ahmed D. Association between sleep and behavioural problems among children with enuresis. J Paediatr Child Health. 2013;49:E160-6. 10.1111/jpc.12017 23198684

[7] 7. Alexopoulos EI, Malakasioti G, Varlami V, Miligkos M, Gourgoulianis K, Kaditis AG. Nocturnal enuresis is associated with moderate-to-severe obstructive sleep apnea in children with snoring. Pediatr Res. 2014;76:555-9. 10.1038/pr.2014.137 25198373

[8] 8. Gür E, Turhan P, Can G, Akkus S, Sever L, Güzelöz S, et al. Enuresis: prevalence, risk factors and urinary pathology among school children in Istanbul, Turkey. Pediatr Int. 2004;46:58-63. 10.1111/j.1442-200X.2004.01824.x 15043666

[9] 9. Freedman DS, Dietz WH, Srinivasan SR, Berenson GS. The relation of overweight to cardiovascular risk factors among children and adolescents: the Bogalusa Heart Study. Pediatrics. 1999;103(6 Pt 1):1175-82. 10.1542/peds.103.6.1175 10353925

[10] 10. Styne DM. Childhood and adolescent obesity. Prevalence and significance. Pediatr Clin North Am. 2001;48:823-54. 10.1016/s0031-3955(05)70344-8 11494639

[11] 11. Erdem E, Lin A, Kogan BA, Feustel PJ. Association of elimination dysfunction and body mass index. J Pediatr Urol. 2006;2:364-7. 10.1016/j.jpurol.2006.05.002 18947637

[12] 12. Weintraub Y, Singer S, Alexander D, Hacham S, Menuchin G, Lubetzky R, et al. Enuresis--an unattended comorbidity of childhood obesity. Int J Obes (Lond). 2013;37:75-8. 10.1038/ijo.2012.108 22828939

[13] 13. Guven A, Giramonti K, Kogan BA. The effect of obesity on treatment efficacy in children with nocturnal enuresis and voiding dysfunction. J Urol. 2007;178(4 Pt 1):1458-62. 10.1016/j.juro.2007.05.165 17706706

[14] 14. Chang SJ, Chiang IN, Lin CD, Hsieh CH, Yang SS. Obese children at higher risk for having overactive bladder symptoms: a community-based study. Neurourol Urodyn. 2015;34:123-7. 10.1002/nau.22532 24273112

[15] 15. Merhi BA, Hammoud A, Ziade F, Kamel R, Rajab M. Mono-symptomatic nocturnal enuresis in lebanese children: prevalence, relation with obesity, and psychological effect. Clin Med Insights Pediatr. 2014;8:5-9. 10.4137/CMPed.S13068 PMC3948714 24653655

[16] 16. Sally S, Zahra. A prospective longitudinal study to estimate the prevalence of obesity in Egyptian children with nocturnal enuresis and the association between body mass index and response to therapy. Egypt J Med Hum Genet. 2016; 18:1-8.

[17] 17. Li H, Ji CY, Zong XN, Zhang YQ. [Height and weight standardized growth charts for Chinese children and adolescents aged 0 to 18 years]. Zhonghua Er Ke Za Zhi. 2009;47:487-92. 19951507

[18] 18. Kuczmarski RJ, Ogden CL, Grummer-Strawn LM, Flegal KM, Guo SS, Wei R, et al. CDC growth charts: United States. Adv Data. 2000;(314):1-27. 11183293

[19] 19. Koff SA. Estimating bladder capacity in children. Urology. 1983;21:248. 10.1016/0090-4295(83)90079-1 6836800

[20] 20. Austin PF, Bauer SB, Bower W, Chase J, Franco I, Hoebeke P, et al. The standardization of terminology of lower urinary tract function in children and adolescents: update report from the Standardization Committee of the International Children’s Continence Society. J Urol. 2014;191:1863-1865.e13. 10.1016/j.juro.2014.01.110 24508614

[21] 21. Barone JG, Hanson C, DaJusta DG, Gioia K, England SJ, Schneider D. Nocturnal enuresis and overweight are associated with obstructive sleep apnea. Pediatrics. 2009;124:e53-9. 10.1542/peds.2008-2805 19564269

[22] 22. Stein RI, Epstein LH, Raynor HA, Kilanowski CK, Paluch RA. The influence of parenting change on pediatric weight control. Obes Res. 2005;13:1749-55. 10.1038/oby.2005.213 16286522

[23] 23. Cummings JM, Rodning CB. Urinary stress incontinence among obese women: review of pathophysiology therapy. Int Urogynecol J Pelvic Floor Dysfunct. 2000;11:41-4. 10.1007/s001920050008 10738933

[24] 24. Diaz Saldano D, Chaviano AH, Maizels M, Yerkes EB, Cheng EY, Losavio J, et al. Office management of pediatric primary nocturnal enuresis: a comparison of physician advised and parent chosen alternative treatment outcomes. J Urol. 2007;178(4 Pt 2):1758-61. 10.1016/j.juro.2007.03.195 17707433

[25] 25. Mellin AE, Neumark-Sztainer D, Story M, Ireland M, Resnick MD. Unhealthy behaviors and psychosocial difficulties among overweight adolescents: the potential impact of familial factors. J Adolesc Health. 2002;31:145-53. 10.1016/s1054-139x(01)00396-2 12127384

[26] 26. Fagot-Campagna A, Pettitt DJ, Engelgau MM, Burrows NR, Geiss LS, Valdez R, et al. Type 2 diabetes among North American children and adolescents: an epidemiologic review and a public health perspective. J Pediatr. 2000;136:664-72. 10.1067/mpd.2000.105141 10802501

[27] 27. Weiss R, Dziura J, Burgert TS, Tamborlane WV, Taksali SE, Yeckel CW, et al. Obesity and the metabolic syndrome in children and adolescents. N Engl J Med. 2004;350:2362-74. 10.1056/NEJMoa031049 15175438

[28] 28. Berenson GS, Srinivasan SR, Bao W, Newman WP 3rd, Tracy RE, Wattigney WA. Association between multiple cardiovascular risk factors and atherosclerosis in children and young adults. The Bogalusa Heart Study. N Engl J Med. 1998;338:1650-6. 10.1056/NEJM199806043382302 9614255

[29] 29. Sakellaropoulou AV, Hatzistilianou MN, Emporiadou MN, Aivazis VT, Goudakos J, Markou K, et al. Association between primary nocturnal enuresis and habitual snoring in children with obstructive sleep apnoea-hypopnoea yndrome. Arch Med Sci. 2012;8:521-7. 10.5114/aoms.2012.28809 PMC3400898 22852010

[30] 30. Beebe DW. Neurobehavioral morbidity associated with disordered breathing during sleep in children: a comprehensive review. Sleep. 2006;29:1115-34. 10.1093/sleep/29.9.1115 17040000

[31] 31. Kaditis AG, Alexopoulos EI, Hatzi F, Kostadima E, Kiaffas M, Zakynthinos E, et al. Overnight change in brain natriuretic peptide levels in children with sleep-disordered breathing. Chest. 2006;130:1377-84. 10.1378/chest.130.5.1377 17099013

[32] 32. Agras WS, Hammer LD, McNicholas F, Kraemer HC. Risk factors for childhood overweight: a prospective study from birth to 9.5 years. J Pediatr. 2004;145:20-5. Erratum in: J Pediatr. 2004;145:424. 10.1016/j.jpeds.2004.03.023 15238901

[33] 33. Kovacevic L, Jurewicz M, Dabaja A, Thomas R, Diaz M, Madgy DN, et al. Enuretic children with obstructive sleep apnea syndrome: should they see otolaryngology first? J Pediatr Urol. 2013;9:145-50. 10.1016/j.jpurol.2011.12.013 22285485

[34] 34. Barone JG, Hanson C, DaJusta DG, Gioia K, England SJ, Schneider D. Nocturnal enuresis and overweight are associated with obstructive sleep apnea. Pediatrics. 2009;124:e53-9. 10.1542/peds.2008-2805 19564269

[35] 35. Sans Capdevila O, Crabtree VM, Kheirandish-Gozal L, Gozal D. Increased morning brain natriuretic peptide levels in children with nocturnal enuresis and sleep-disordered breathing: a community-based study. Pediatrics. 2008;121:e1208-14. 10.1542/peds.2007-2049 18450864

[36] 36. Kurt O, Yazici CM, Paketci C. Nocturnal enuresis with spina bifida occulta: Does it interfere behavioral management success? Int Urol Nephrol. 2015;47:1485-91. 10.1007/s11255-015-1047-4 26149636

[37] 37. Önol FF, Guzel R, Tahra A, Kaya C, Boylu U. Comparison of long-term efficacy of desmopressin lyophilisate and enuretic alarm for monosymptomatic enuresis and assessment of predictive factors for success: a randomized prospective trial. J Urol. 2015;193:655-61. 10.1016/j.juro.2014.08.088 25158273

[38] 38. Ferrara P, Del Volgo V, Romano V, Scarpelli V, De Gara L, Miggiano GA. Combined Dietary Recommendations, Desmopressin, and Behavioral Interventions May Be Effective First-Line Treatment in Resolution of Enuresis. Urol J. 2015;12:2228-32. 26341763

